# Diagnosis and Treatment of Eclampsia

**DOI:** 10.3390/jcdd11090257

**Published:** 2024-08-23

**Authors:** Vasiliki Katsi, Asimenia Svigkou, Ioanna Dima, Konstantinos Tsioufis

**Affiliations:** 1 Cardiology Department, School of Medicine, Hippokration General Hospital, National and Kapodistrian University of Athens, 157 72 Athens, Greece; vkkatsi@yahoo.gr (V.K.); kptsioufis@gmail.com (K.T.); 2 Independent Researcher, 163 42 Athens, Greece; 3 Cardiology Department, Helena Venizelou Hospital, 115 21 Athens, Greece; ioadim2006@yahoo.gr

**Keywords:** hypertensive disorder of pregnancy, preeclampsia, magnesium sulfate, labetalol, hydralazine, nifedipine, delivery

## Abstract

Hypertensive disorders of pregnancy affect approximately 5% to 10% of pregnant women. Eclampsia is a serious hypertensive disorder that is primarily characterized by the onset of grand mal seizure activity in the absence of other causative conditions. While eclampsia is diagnosed clinically, laboratory tests are recommended to assess for complications. Treatment strategies for eclampsia focus on controlling seizures and managing hypertension. Acute care during a seizure is critical because of the need for immediate medical interventions, including the management of the airway, breathing, and circulation, as well as ensuring the safety of the patient during convulsions. Magnesium sulfate is the preferred anticonvulsant drug. Care must be taken during administration to prevent magnesium toxicity. Antihypertensive drugs used in eclampsia include labetalol, hydralazine and nifedipine. The definitive treatment of eclampsia is delivery. Close monitoring of both mother and fetus is important to identify any indications for delivery. The timing and mode of delivery depend on obstetric indications, the severity of eclampsia, the gestational age of the fetus, and the overall clinical status of the patient. Neuraxial anesthesia is the anesthesia of choice for conscious, seizure-free, and with stable vital signs women undergoing cesarean section.

## 1. Introduction

Hypertensive disorders of pregnancy affect approximately 5% to 10% of pregnant women. Preeclampsia and eclampsia account for approximately half of these cases worldwide [1]. These conditions are responsible for up to 14% of all maternal deaths worldwide. The rate of eclampsia and the number of maternal deaths from hypertension during pregnancy differ between developed and developing countries, with developing countries having an increased rate [2]. The word eclampsia is borrowed from New Latin eclampsia, from Ancient Greek éklampsis, (“sudden development, violent onset”), from eklámpō, (“to shine”), and -sis, a nominal suffix [3].

One of the primary pathological outcomes of uteroplacental ischemia in preeclampsia is the release of pro-inflammatory cytokines and anti-angiogenic factors into the maternal circulation, leading to endothelial dysfunction. Endothelial dysfunction directly leads to disruption of the blood–brain barrier (BBB) by increasing its permeability, resulting in an imbalance of ions, neurotransmitters, and metabolic products in the interstitial fluid [4]. The anti-angiogenic imbalance may also perturb the myogenic tone of the vascular smooth muscle, and uncontrolled vasoconstriction may cause abnormalities in the autoregulation of cerebral circulation [5]. Since maintaining the homeostasis of the brain environment is crucial, both hypoperfusion and hyperperfusion may cause significant harm. Insufficient cerebral blood flow may result in regions of ischemia and brain injury [6]. Hyperperfusion, on the other hand, combined with BBB dysfunction may result in vasogenic edema. Eclamptic seizures are the result of edema and/or dysregulation of the neuronal microenvironment due to BBB breakdown [5].

According to a meta-analysis of the genome-wide association of mothers, there is a genetic predisposition to eclampsia. Sequence variants in the maternal genome previously known to be linked to blood pressure have been identified. Furthermore, the polygenic risk score for blood pressure (BP-PRS) appears to be associated with preeclampsia [7]. The polygenic risk score indicates an individual’s genetic susceptibility to a disease influenced by multiple genetic variants. It is calculated as a weighted sum of the risk alleles identified in genome-wide association studies that are associated with the disease. Kivioja et al. observed that women with BP-PRS > 95th percentile had an increased risk for preeclampsia, recurrent preeclampsia in further pregnancies, and preeclampsia with severe features [8]. A history of preeclampsia significantly increases the risk of recurrence in subsequent pregnancies by seven to ten times, with the recurrence typically occurring at a later gestational age [9].

## 2. Diagnosis of Eclampsia

### 2.1. Clinical Manifestations

Preeclampsia is defined as the new onset of hypertension (systolic blood pressure (SBP) > 140 mmHg or diastolic blood pressure (DBP) > 90 mmHg) at or after 20 weeks of pregnancy and the coexistence of one or more of the following new-onset conditions: significant proteinuria, maternal organ dysfunction, or uteroplacental dysfunction [3]. Eclampsia is a convulsive condition and the most severe manifestation of preeclampsia [10]. It is defined as the new onset of tonic–clonic seizure activity (usually lasts 60–90 min) and/or coma in the absence of other causative conditions [11]. In some cases, either hypertension or proteinuria are absent [12]. Studies have shown that over one-third of eclampsia cases occur suddenly without any preceding signs or symptoms of preeclampsia. However, up to 83% of eclampsia cases are linked to prodromal symptoms before seizures occur [13]. These include neurologic symptoms such as severe and frontal or atypical headache, generalized edema, vision disturbances, amnesia, and non-neurologic symptoms such as right upper quadrant or epigastric pain [14]. Vision changes may include blurry vision, scotoma, photopsia, diplopia, and transient blindness. Unfortunately, these symptoms cannot definitively confirm or exclude the diagnosis, and in many cases, the patient may be unable to provide a history due to being altered or actively seizing [15]. The onset of eclamptic convulsions can be in the antepartum, intrapartum, or postpartum period. Although the definition includes symptoms and signs past 20 weeks of gestation, almost all cases of antepartum eclampsia will occur after 28 weeks of gestation [16]. Eclampsia can occur before 20 weeks of gestation in patients with renal disease, molar pregnancy, or multiple gestations [17].

### 2.2. Physical Examination

Physical examination findings in eclampsia can vary. Common findings include hypertension, tachycardia, tachypnea, mental status changes, edema, clonus, and hyperreflexia [18]. Accurate blood pressure measurement with an appropriately sized cuff is crucial, although the severity of hypertension does not predict eclampsia. About 20% of eclampsia cases occur with mild hypertension, and blood pressure may be normal in 25% of patients [15].

### 2.3. Laboratory Evaluation

Although eclampsia is a clinical diagnosis, laboratory tests are recommended to evaluate the disease severity, complications, and other possible or concurrent causes. Once the initial evaluation is performed, appropriate management is then guided. Laboratory evaluation includes blood cell count, renal and liver function panels, electrolytes, glucose, coagulation panel, fibrinogen, lactate dehydrogenase (LDH), uric acid, and urinalysis [19]. Thrombocytopenia, elevated creatinine, elevated aminotransferases, elevated LDH and uric acid, and proteinuria may be revealed [13]. HELLP syndrome, whose acronym is derived from the symptomatic triad “Hemolysis, Elevated Liver enzymes, and Low Platelet count”, represents a complication of severe preeclampsia and is diagnosed on the basis of biochemical laboratory evidence. Its diagnosis should be ruled out because of the high mortality during pregnancy and the postpartum period [20].

In addition to eclamptic seizures, preeclampsia is associated with other neurological complications such as cerebral venous sinus thrombosis (CVST), reversible cerebral vasoconstriction syndrome (RCVS), posterior reversible encephalopathy syndrome (PRES), subarachnoid hemorrhage (SAH), intracerebral hemorrhage (ICH), and arterial ischemic stroke [21]. Neurological complications during pregnancy and the postpartum period often overlap significantly, making it difficult to distinguish between them due to their many similarities. Therefore, patients with new-onset seizures, altered mental status, or focal neurological deficits should undergo non-contrast head computed tomography (CT) or magnetic resonance imaging (MRI) to check for intracranial issues [22]. Furthermore, patients with cardiovascular or respiratory symptoms should undergo an electrocardiogram and chest radiography to evaluate for dysrhythmias, right heart strain, and pulmonary edema and pneumonia, respectively [19].

## 3. Management of Eclampsia

**Figure 1:**
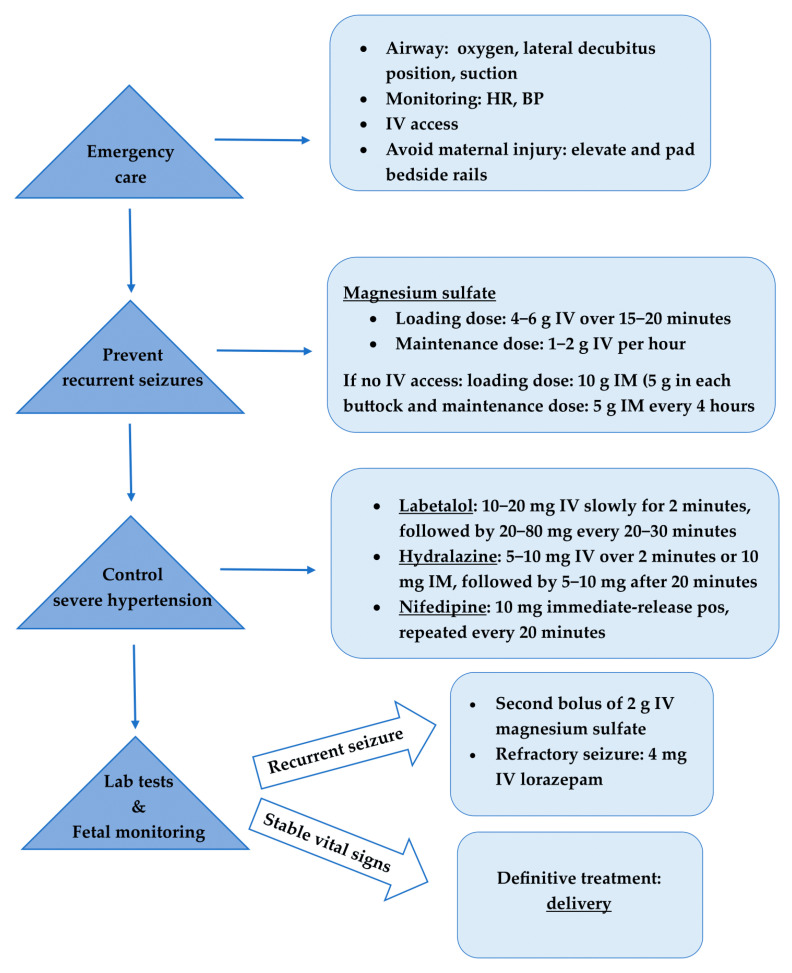
Treatment algorithm for eclamptic seizure. HR, heart rate; BP, blood pressure; IV, intravenous; IM, intramuscular.

The management of eclampsia uses a team-based approach that involves obstetricians, anesthesiologists, labor and delivery nurses. Eclamptic women should only be cared for in hospitals with adequate medical ICUs and transported by ambulance with medical personnel present for proper management in case of subsequent convulsions [23]. Actions to manage eclampsia include emergency care during the seizure to ensure the health and safety of both mother and fetus, medication to stop eclampsia seizures and their recurrence, and antihypertensive drugs to control hypertensive crises [24]. The definitive treatment of eclampsia is delivery of the fetus as soon as the woman is stabilized [2,13,18]. Therefore, eclamptic women require immediate obstetric consultation and admission to an equipped labor and delivery unit. If there is a risk of premature birth or fetal disturbance, the advice of a pediatrician or neonatologist is also necessary [18]. While the management of eclampsia varies in different regions, the therapeutic approach to eclampsia in most parts of the world typically follows the algorithm presented in Figure 1.

### 3.1. Supportive Care during a Seizure

As soon as a convulsion is clinically recognized, foundational emergency treatment should be provided for stabilization. The first step is to assess and establish the airway and ensure oxygenation [25]. Administering oxygen therapy is crucial for maintaining proper saturation levels and treating hypoxemia that occurs from hypoventilation during the seizure activity. An oxygen flow rate of over 8−10 L/min through a facial mask provides more than 60% oxygen concentration in the breathing mixture and reduces the risk of respiratory acidosis. If oxygen saturation falls below 93% by pulse oximetry, it is necessary to assess arterial blood gas and administer bicarbonate in case of acidosis [26]. After the seizure has ended, the patient usually starts to breathe normally and does not need supplementary oxygen [27]. However, in women who have repetitive seizures, aspiration pneumonia or pulmonary edema may lead to maternal hypoxia [2]. The patient should be placed in the lateral decubitus position to decrease the risk of aspiration of gastric contents into the respiratory system and improve uterine blood flow by relieving obstruction of the vena cava by the gravid uterus [28]. Furthermore, the oral secretions should be suctioned as needed [29]. Cardiac monitoring is deemed necessary to ensure the ongoing assessment and surveillance of cardiac function and rhythm [25]. During convulsion, the aim is to prevent maternal injury during the seizure. For this reason, bedside rails should be elevated and padded [29]. If possible, an intravenous (IV) line with a 16- to 18-gauge catheter should be already present before the seizure to administer the necessary medication and to maintain the fluid and electrolyte balance [18]. Fluid management is critical in patients with eclampsia because volume expansion can lead to fluid overload and pulmonary edema, and fluid restriction can worsen tissue hypoperfusion and renal function [30]. Therefore, fluid administration should be individualized according to clinical assessment and, where available, noninvasive hemodynamic measurements, such as lung ultrasound, transthoracic echocardiography, or pulse wave-form monitors [31].

### 3.2. Treatment of Convulsions

The next step in the management of eclampsia is the prevention of recurrent seizures [2]. Magnesium sulfate is the drug of choice for preventing recurrent convulsions in women with eclampsia and preventing seizures in women with preeclampsia, as shown in two large clinical studies [32,33]. In 1995, the Eclampsia Trial Collaborative Group reported that magnesium sulfate significantly reduced the risk of recurrent seizures compared to other antiepileptic agents [32]. In 2002, the Magpie trial demonstrated that women with severe preeclampsia who received magnesium sulfate had a 58% lower risk of developing eclampsia compared to those given a placebo [33]. Although the effectiveness of magnesium sulfate in treating eclampsia has been established, its mechanism of action remains unclear [34]. One possible mechanism is the improvement of both the placental circulation and the vascular response to dilators by enhancing the nitric oxide synthase (NOS) activity and maintaining the balance between angiogenic and antiangiogenic growth factors [35]. Apart from being a vasodilator, magnesium also acts as a central anticonvulsant by increasing the seizure threshold after inhibiting N-methyl-d-aspartate (NMDA) receptors [34]. Furthermore, magnesium, as a calcium antagonist, can affect the cerebral endothelium by protecting the BBB and reducing the formation of cerebral edema [36].

Magnesium sulfate is the first-line treatment to control eclamptic seizures. A loading dose of 4 or 6 g should be administered IV over 15 to 20 min [37]. Convulsions usually stop after the loading dose. A maintenance dose of 1 or 2 g per hour should be administered as a continuous IV solution in order to prevent recurrent seizures. Studies have shown that 2 g are more likely to produce the mean therapeutic concentration of magnesium sulfate with fewer fluctuations during the period of administration compared with 1 g per hour [38]. Women with a body mass index (BMI) ≥ 35 kg/m^2^ are significantly more likely to reach therapeutic serum magnesium concentrations when given a 6 g IV loading dose followed by a 2 g per hour IV maintenance dose, compared to those receiving a 4 g IV loading dose followed by a 1 g per hour IV maintenance dose [39]. In patients who do not have available intravenous access, magnesium sulfate can be given by intramuscular (IM) injection. The loading dose is 10 g (5 g in each buttock), and the maintenance dose is 5 g every 4 h [40]. The serum level fluctuates more with this regimen than with continuous IV solution [38]. Approximately 10% of women experience a second seizure after receiving magnesium sulfate. Thus, a second bolus of 2 g of IV magnesium sulfate can be administered to these women for 3 to 5 min [41]. In rare cases of recurrent convulsions, despite adequate and therapeutic doses of magnesium sulfate, the recommended treatment is 4 mg of lorazepam administered IV within 3−5 min [2].

Magnesium sulfate is generally a safe drug for women. However, as with any medication, it may cause several side effects. The main symptoms are hypotension secondary to reductions in systemic vascular resistance, facial flushing, flushing at the injection site, increased warmth and sweating due to the peripheral vasodilatory effects of magnesium, visual disturbances, chest pain, and nasal stuffiness [42]. For women who are treated with cardiac glycosides/digitalis, magnesium sulfate should be administered with caution, as digoxin can induce hypermagnesuria with several important clinical consequences [43]. Additionally, the concurrent use of magnesium sulfate and depressants may result in enhanced central nervous system depression [44]. Magnesium sulfate should ideally be infused two hours before fetal delivery due to possible interaction with neuromuscular blocking medication given intraoperatively [45]. In patients with neuromuscular diseases like myasthenia gravis, neuromuscular function can deteriorate even at lower medication concentrations. Therefore, the use of magnesium sulfate is contraindicated in these diseases [46]. Other contraindications include severe hypocalcemia, complete heart block, and myocarditis [47].

The therapeutic blood levels of magnesium are 4–7 mEq/L [48]. As levels continue to rise, findings of toxicity start to manifest. Patients develop muscle weakness and loss of deep tendon reflexes at serum levels of 8–10 mEq/L, while respiratory depression occurs at 15 mEq/L [49]. Additionally, patients experience ECG changes (prolonged PR interval and widened QRS) at 5–10 mEq/L, signs of abnormal conductivity surface as sinoatrial (SA) or atrioventricular (AV) node block at 15 mEq/L, and a high risk of cardiac arrest at 20 mEq/L or higher [50]. However, there is no need for routine monitoring of magnesium levels except for women with renal impairment or electrolyte imbalance. Magnesium is excreted almost exclusively by the kidneys. Consequently, there is an increased risk of magnesium toxicity under these conditions [51]. The loading dose does not change in women with a serum creatinine >1.2 mg/dl or oliguria (<30 mL urine output per hour for more than 4 h). However, the maintenance dose should be different, and a dose of only 1 g per hour should be administered [52]. It is also necessary to monitor reflexes, creatinine function, and urine output during the administration of magnesium to prevent toxicity [53]. The maintenance dose should be given only if tendon reflexes are present, the respiratory rate is >12/min, the urine output exceeds 100 mL in 4 h, and therapeutic blood levels are within the normal range [54].

At the first sign of toxicity, both magnesium sulfate administrations should be discontinued and excess magnesium removed from the body. In patients with normal kidney function, this can be achieved with intravenous diuretics, while dialysis treatment is necessary for those with impaired kidney function [55]. In severe cases, an antidote can be used to displace and neutralize the effects of magnesium. The antidote is 10 mL of an IV solution of 10% calcium gluconate for 5 min [54]. In addition to the antidote, emergent intubation may be required in women with an increased risk of respiratory depression [49]. Calcium gluconate should be given with caution because it may precipitate heart blocks [44]. In women with impaired renal function or signs concerning magnesium toxicity, the measurement of magnesium in blood is recommended every 4 to 6 h. The infusion should be paused if the serum level exceeds 8 mEq/L and restarted at a lower rate when the serum level decreases to <7 mEq/L [47]. Importantly, the administration of magnesium should be continued for 24 h after delivery or at least 24 h after the last convulsion [56].

Even though magnesium sulfate is not an anticonvulsant and is not used in other seizure conditions, it remains the preferred medication for controlling eclamptic seizures [57]. It shows greater effectiveness than other medications (i.e., benzodiazepines, phenytoin) [58,59]. However, these antiepileptic medications can be used for patients in whom magnesium at therapeutic doses is ineffective or if there are contraindications for the use of magnesium sulfate [60]. Approximately 10% of patients do not respond to magnesium and require an IV administration of diazepam at a dose of 5–10 mg or phenytoin at a dose of 250 mg (up to 750–1250 mg based on the body mass) within 12 h. The therapeutic level of phenytoin is 12 mg/mL [24]. Women with myasthenia gravis should be treated with levetiracetam or valproic acid because magnesium and phenytoin cause muscle weakness that can be followed by a myasthenia crisis [37].

### 3.3. Treatment of Hypertension

Blood pressure control is the next step after anticonvulsant therapy [61]. Severe hypertension (SBP ≥ 160 mmHg and/or DBP ≥ 110 mmHg) should be treated after magnesium infusions to prevent ICH and pulmonary edema, the two major causes of mortality from eclampsia, both of which are related to hypertension [62]. Women with eclampsia or preeclampsia have a corresponding 9.23 times higher risk of ICH than those without these diseases [63]. Vascular changes of ICH in eclampsia may result from arteriolar dysfunction, where impaired autoregulation is unable to manage acute increases in blood pressure [64]. Hypertension continuously weakens blood vessel walls, eventually causing them to rupture and bleed [65]. Pulmonary edema may also occur in approximately 3–5% of women with hypertensive disorders of pregnancy, with 70% of cases occurring in the postpartum period. The etiology of pulmonary edema is most likely a combination of reduction in oncotic pressure secondary to preeclampsia-related hypoproteinemia, damage of pulmonary endothelium leading to increased capillary permeability, inadvertent fluid administration, impaired cardiac function, and increased afterload due to severe hypertension [30].

The aim is to maintain the SBP between 140 and 160 mmHg and the DBP between 90 and 110 mmHg [66]. Caution must be taken not to reduce blood pressure too drastically due to the risk of a sudden decrease in blood flow to maternal organs, including the uteroplacental circulation, which can lead to fetal hypoxia or even intrauterine fetal death [18]. Mean arterial blood pressure should be decreased by 15–20% in patients with severe hypertension [67]. First-line pharmacological treatments for hypertension in eclamptic women include labetalol, hydralazine, and nifedipine [68]. IV administration is necessary in unconscious patients with eclampsia. The choice of an antihypertensive drug should be individualized, taking into account the clinical condition of the patient, the availability of the drug, the effects of the drug on the developing fetus, and the experience of the medical staff [69].

Labetalol, as a mixed alpha-adrenergic and beta-adrenergic antagonist, causes vasodilation and a reduction in heart rate [70]. An initial dose of 10–20 mg IV slowly for 2 min, followed by 20–80 mg every 20–30 min to a maximum dose of 300 mg in 24 h, is recommended until the target blood pressure is reached [71]. The mechanism of action of labetalol involves reducing peripheral vascular resistance without compromising blood flow to the brain or peripheral, coronary, or renal systems. As a nonselective beta-blocker, it should be given with caution in women with mild and moderate asthma and is contraindicated in women with severe asthma. Other contraindications include congestive heart failure, left ventricle dysfunction, and AV heart block due to the risk of bradycardia [72]. The risk of neonatal bradycardia and hypoglycemia may also be increased with labetalol [73]. Caution should also be exercised in women with a history of impaired liver function, as severe hepatocellular injury is a recorded, very rare complication. Hepatotoxicity may be confused with HELLP syndrome. Most cases of labetalol-induced liver toxicity are reversible, but deaths have been reported [74].

Hydralazine causes dilation of blood vessels, leading to a reduction in peripheral vascular resistance and blood pressure [75]. An IV bolus of 5–10 mg over 2 min or 10 mg IM, followed by 5–10 mg after 20 min, up to a total dose of 20 mg IV or 30 mg IM should be given if the SBP is greater than 160 mmHg or the DBP is greater than 110 mmHg [76]. Hydralazine should not be used in the presence of tachycardia greater than 100 bpm because of short-term adverse effects in the form of reflex tachycardia and heart pounding. Labetalol is preferable to hydralazine because it has a quicker onset of action (approximately 5 min) and a lower risk for reflex tachycardia [77]. A sudden drop in blood pressure may also occur, leading to a significant reduction in uteroplacental blood flow, disrupted fetal heart rate, and fetal bradycardia [24]. Cesarean section rates have increased secondary to fetal distress caused by these pulse disruptions [78]. Besides maternal hypotension, other possible side effects include headaches, chest pain, dizziness, reduced urine output, and a high incidence of placental abruption [79].

Alternatively, oral nifedipine may be administered, particularly when IV access has not yet been initiated. Nifedipine is a dihydropyridine calcium channel blocker that promotes vasodilation and reduces systemic vascular resistance [80]. A starting dose of 10 mg immediate-release nifedipine should be given, repeated every 20 min up to a maximum daily dose of 180 mg [81]. Nifedipine should be administered as a tablet or capsule, each of which should be swallowed whole, not bitten or punctured [82]. Nifedipine should not be given sublingually because of possible irreversible severe hypotension with consequent severe maternal and perinatal morbidity and mortality [81]. Tachycardia, flushing, peripheral edema, dizziness, and headache are the main adverse effects [80]. Furthermore, combining calcium channel blockers with magnesium sulfate requires caution because it could cause neuromuscular blockade or uterine relaxation, although these effects are very rare [83].

According to a meta-analysis of 11 studies comparing the effectiveness of antihypertensive drugs, oral nifedipine was more effective at reducing the risk of persistent high blood pressure during pregnancy compared to IV hydralazine and IV labetalol. There were no significant differences in the rates of maternal hypotension, adverse effects, or outcomes for both the mother and fetus [84]. Furthermore, a network meta-analysis (17 trials, *N* = 1591 women) comparing one first-line antihypertensive agent with another first-line agent for the treatment of severe hypertension in pregnancy revealed that nifedipine was more effective in the treatment of severe hypertension than IV hydralazine [85]. Zulfeen et al. showed that although both labetalol and nifedipine were effective in controlling blood pressure, blood pressure was decreased more rapidly with nifedipine. Therefore, oral nifedipine may be a preferable option due to its easy oral administration and consistent dosing regimen [71]. If neither IV access nor oral nifedipine is available, a 200 mg dose of labetalol can be given orally and repeated within 30 min [66].

Second-line alternatives for the treatment of resistant hypertension include the use of nicardipine or esmolol via infusion pumps [29]. Sodium nitroprusside or nitroglycerine should be reserved for extreme emergencies and used for the shortest amount of time [86]. They contain cyanide and have the potential to cause maternal, fetal, and neonatal cyanide and thiocyanate toxicity and increased intracranial pressure, with the potential worsening of cerebral edema in women [87]. Diuretics are not often used in eclampsia, a condition characterized by lower plasma volume, because they may further exacerbate volume depletion and cause reactive vasoconstriction. They are used only in cases of pulmonary edema prior to delivery [88]. Methyldopa, an alpha-2 adrenergic agonist, is a safe antihypertensive medication during pregnancy but is less effective than beta-blockers and calcium channel blockers in controlling severe hypertension [89]. Angiotensin-converting enzyme inhibitors and angiotensin II receptor blockers are recognized teratogens that cause adverse effects on the developing fetal renal system, such as emerging kidney injury and oligohydramnios [90]. They have also been associated with skull anomalies, and thus they are contraindicated throughout pregnancy [91].

### 3.4. Management of an Eclamptic Patient after a Convulsion

Once eclamptic seizure has stopped, close monitoring of both mother and fetus is essential to identify indications for delivery. Maternal monitoring includes respiratory rate and oxygenation, heart rate and blood pressure, fluid intake and urine output, and neurologic status for signs of increased intracranial pressure or bleeding [92]. In cases of signs of pulmonary edema or oliguria/anuria, monitoring of pulmonary arterial blood pressure is indicated [18]. If initial laboratory results are normal, no repeat is needed after the seizure. Conversely, if results have shown thrombocytopenia (<100,000/μL) or elevated creatinine (>0.1 mg/dL), laboratory evaluation should be repeated in 6 h. Platelet count and kidney function need to be stabilized before delivery [2]. In a stable, nonbleeding woman, a platelet transfusion is recommended if the platelet count is <50,000/μL before cesarean or <20,000/μL for vaginal delivery [93]. Antenatal corticosteroids are also recommended for pregnant women between 24 0/7 weeks and 33 6/7 weeks of gestation because they help lung maturation and generally improve neonatal outcomes. A dose of 12 mg IM betamethasone or 6 mg IM dexamethasone should be administered in anticipation of emergent delivery [94].

Fetal monitoring may reveal changes in fetal heart rate. Ambia et al. studied 31 women who experienced 34 eclamptic seizures and found that fetal heart rate decelerations were observed in 79% of cases; the mean duration of bradycardia was 5.80 ± 2.98 min with a range of 2–15 min; and the interval from the onset of the seizure to the fall in the fetal heart rate was 2.7 ± 1.6 min. Furthermore, they observed that half of the fetuses with prolonged decelerations developed tachycardia following bradycardia [95]. These heart rate abnormalities are related to maternal lactic acidosis caused by intense vasospasm and uterine hyperactivity during the convulsion, leading to reduced uteroplacental blood flow and fetal hypoxia [96]. Despite these fetal heart rate changes, emergent operative intervention is not indicated for this transient bradycardia because of its spontaneous resolution. The primary focus should be on maternal oxygenation and stabilization [95]. Growth-restricted and preterm fetuses may have a prolonged recovery time after a seizure. Continued uterine hyperactivity and persistent fetal bradycardia may indicate placental abruption, which needs further management [18].

### 3.5. Delivery

Delivery is the definitive treatment of eclampsia when the acute phase of the seizure has passed. No attempt should be made to deliver the infant as long as the woman is unstable [2,13]. The aim of stabilizing is the best assessment, followed by determining the safest mode of delivery within a reasonable time [97]. The mode of delivery should depend on obstetric indications such as gestational age, fetal condition, the presence of labor, and the cervical Bishop score [2]. Vaginal delivery and even the possibility of labor induction or acceleration are recommended in women after an eclamptic seizure, provided they are conscious, stable, oriented, have confirmed fetal well-being, and have a favorable cervix (Bishop score 6 or greater), with labor expected to be complete within a few hours [98]. It is the most common mode and is preferable from a maternal standpoint [18]. Induction with an IV oxytocin infusion should be started once both maternal and fetal status are stable and as long as the patient is in the active phase within 24 h [99].

On the other hand, patients with an unfavorable cervix (Bishop score < 5) and a gestational age of 30 weeks or less have a very low (<10%) success rate in induction of labor [2]. Under these circumstances, and especially in patients without contractions, emergency labor via cesarean section without peritonization with the Douglas’ drainage pouch is preferable once the patient’s condition is stabilized due to the severe and unpredictable consequences associated with vaginal delivery for both mother and fetus [98]. Other indications for cesarean delivery are the absence of end-diastolic blood flow on Doppler scans and reversed end-diastolic blood flow on Doppler scans [99]. During cesarean section, the patient should be placed on the left lateral side, which helps reduce compression on the inferior vena cava. Therefore, the operating table should be slightly tilted by 15° [100].

According to several observational studies across the world, there is an increasing trend of delivering by cesarean section instead of inducing labor and delivering vaginally [101,102,103]. It is possible that the decision of mode of termination is influenced by the recommendations of some authorities that all women with eclampsia should be delivered within 12 h following seizure(s) [104]. When delivery is not anticipated within 6 h or when there is an unfavorable cervix, cesarean section is associated with improved maternal and perinatal outcomes [105]. Otherwise, an induction is associated with a prolonged intrapartum course and thus intrapartum complications. The most frequent intrapartum complications are fetal growth retardation, nonreassuring fetal heart rate patterns, and placental abruption [18].

### 3.6. Analgesia–Anesthesia

Maternal pain relief for women who undergo induction of labor can be provided by epidural analgesia, although combined-spinal epidural techniques have also been used [106]. Effective analgesia helps stabilize blood pressure by dampening hypertensive responses associated with painful uterine contractions. Furthermore, neuraxial analgesia can increase intervillous blood flow and improve placental gas exchange by reducing uterine artery resistance [107]. The presence of an epidural catheter allows epidural analgesia to be easily extended to provide anesthesia for an emergency cesarean section. Epidural catheterization can be placed in advance at an appropriate time, even before labor begins or coagulation deteriorates in women with difficult airways, obesity, or other comorbidities [108]. If neuraxial analgesia is contraindicated, patient-controlled analgesia with remifentanil can be used. Although a large amount crosses the placenta, it is quickly metabolized, minimizing the risk of neonatal respiratory depression [106].

Neuraxial anesthesia is the choice in conscious, seizure-free, with stable vital signs and no signs of increased intracranial pressure (ICP) among women undergoing cesarean section [109]. Low-dose hyperbaric bupivacaine (7.5 mg) with 25 μg fentanyl offers sufficient anesthesia for a cesarean section. Patient refusal, placental abruption, coagulopathy, or severe thrombocytopenia (<50,000/mL) are contraindications to regional anesthesia [110]. According to the American College of Obstetricians and Gynecologists, 10% of pregnancies are complicated with thrombocytopenia. Of these, 80% are classified as gestational thrombocytopenia, with the majority having platelet counts greater than 75,000–80,000/mL, a level at which performing a neuraxial block is considered acceptable [111]. Thrombocytopenia has been considered a relative or even absolute contraindication to neuraxial techniques due to the risk of epidural hematoma. The risk of epidural hematoma is <0.2% with a platelet count greater than 70,000/mL [112]. When platelet counts are within the range of 50,000–75,000/mL, an individual assessment is necessary, taking into account patient risks and coagulation tests [110]. Spinal anesthesia may be used with caution because of the risk of total sympathetic blockade, which can lead to maternal hypotension and uteroplacental insufficiency. Small doses of phenylephrine could be used as a preferred vasoconstrictor for preventing and treating hypotension, while additional intravenous fluid is infused judiciously [113]. Spinal anesthesia is safer than general anesthesia in terms of stable vital signs [114]. However, general anesthesia has been considered the optimal choice in unconscious, obtunded women with evidence of increased ICP. Anesthesia is achieved with opioids such as fentanyl, alfentanil, and remifentanil, as well as relaxants and hyperventilation [107]. General anesthesia increases the risk of aspiration and airway edema, leading to difficult intubation if needed. Patients with airway or laryngeal edema may require awake intubation under fiberscope observation [110].

### 3.7. Postpartum Eclampsia

Whether postpartum preeclampsia/eclampsia represents a separate entity from preeclampsia/eclampsia with antepartum onset is still debated [115]. Postpartum preeclampsia/eclampsia typically presents within 48 h after delivery, with the highest risk extending through the first week postpartum [116]. Late postpartum preeclampsia, on the other hand, should be considered in women who develop new-onset hypertension between 48 h and 6 weeks after delivery [117]. Forty-eight hours has traditionally been used because it typically covers immediate postpartum changes. Although the timing is not explicitly defined, it is commonly referred to in expert discussions and existing literature [118]. It has been observed that approximately 20–30% of eclamptic seizures occur during the postpartum period [2]. Less commonly, eclampsia has been reported in 10–15% of women with delayed-onset postpartum preeclampsia, often present with neurological symptoms [119]. Therefore, magnesium sulfate is recommended in new-onset postpartum preeclampsia with any neurological symptoms, particularly in the first week after delivery [120]. Similar to antepartum eclampsia, management of severe hypertension is also important due to the increased risk of maternal morbidity [121]. As there are no fetal concerns post-delivery, treatment can be initiated at a lower threshold, such as 150/100 mmHg, to prevent severe hypertension [122]. First-line antihypertensive agents used are similar to those used during pregnancy, including IV labetalol, IV hydralazine, and oral nifedipine [123].

## 4. Conclusions

Because eclampsia is a rare but life-threatening condition, protocols should be in place for the diagnosis and management of women who experience seizures. Evaluation of clinical symptoms and blood pressure monitoring remain the most effective methods for diagnosing eclampsia, thus allowing early intervention. Acute care during a seizure is crucial and involves immediate medical interventions and essential monitoring protocols, including the oversight of the airway, breathing, and circulation, to ensure patient safety during convulsions. Treatment strategies for eclampsia prioritize seizure control and high blood pressure management. Magnesium sulfate is the preferred anticonvulsant drug. Although its mechanism of action remains unclear, careful administration is necessary to prevent magnesium toxicity. First-line antihypertensive agents for treating eclamptic women include IV labetalol, IV hydralazine, and oral nifedipine. Postseizure management encompasses monitoring and addressing potential complications. Following the acute seizure phase, the definitive treatment for eclampsia is delivery. The mode of delivery depends on obstetric indications, gestational age, and the overall clinical status of the patient, with a delicate balance of risks to the mother and fetus. Neuraxial anesthesia is the preferred choice for conscious and with stable vital signs women.

## 5. Future Directions

The reasons why some patients develop eclampsia and not others remain unknown. A better understanding of the pathophysiology of preeclampsia is crucial for the development of new methods for the diagnosis, treatment, and prevention of eclampsia [24]. It is essential to evaluate the cost-effectiveness and clinical utility of postpartum follow-up for women with preeclampsia, including the frequency of blood pressure monitoring, symptom assessment, and prevention of eclampsia, an important maternal complication [2]. The optimal duration for postpartum magnesium sulfate prophylaxis in women with severe preeclampsia and whether women with delayed-onset preeclampsia (>48 h) would benefit from magnesium sulfate prophylaxis remain a source of debate [124]. Furthermore, although eclampsia is associated with long-term neurological and neurocognitive changes, the risk to maternal health should be further evaluated [125]. The risk of recurrence and obstetrical complications in subsequent pregnancies should also be assessed [2]. Finally, future directions include determining postpartum eclampsia incidence and risk factors, as well as developing evidence-based management algorithms for postpartum eclampsia because it is still a significantly understudied disease [115].

## Data Availability

Not applicable.
